# Fabrication of Multifocal Microlens Array by One Step Exposure Process

**DOI:** 10.3390/mi12091097

**Published:** 2021-09-11

**Authors:** Wei Yuan, Yajuan Cai, Cheng Xu, Hui Pang, Axiu Cao, Yongqi Fu, Qiling Deng

**Affiliations:** 1 School of Physics, University of Electronic Science and Technology of China, Chengdu 610054, China; 201922120308@std.uestc.edu.cn (W.Y.); 201922120310@std.uestc.edu.cn (C.X.); 2 Institute of Optics and Electronics, Chinese Academy of Sciences, Chengdu 610209, China; ph@ioe.ac.cn (H.P.); dengqiling@ioe.ac.cn (Q.D.); 3 School of Information Science and Technology, Southwest Jiao Tong University, Chengdu 610031, China; yw13469@163.com

**Keywords:** integrated micro-optical system, mask moving exposure, microlens array, multifocal

## Abstract

Microlenses can be widely used in integrated micro-optical systems. However, in some special applications, such as light field imaging systems, multifocal microlens arrays (MLA) are expected to improve imaging resolution. For the fabrication of multifocal MLA, the traditional fabrication method is no longer applicable. To solve this problem, a fabrication method of multifocal MLA by a one step exposure process is proposed. Through the analyses and research of photoresist AZ9260, the nonlinear relationship between exposure dose and exposure depth is established. In the design of the mask, the mask pattern is corrected according to the nonlinear relationship to obtain the final mask. The continuous surface of the multifocal MLA is fabricated by the mask moving exposure. The experimental results show that the prepared multifocal MLA has high filling factor and surface fidelity. What is more, this method is simple and efficient to use in practical applications.

## 1. Introduction

Microlens arrays (MLA), as increasingly important optical elements, have the characteristics of small volume, light weight and compact structure, and they provide a general method for the integration of micro optical systems. MLA are widely used in various fields, such as stereoscopic display [1], beam shaping [2], three-dimensional (3D) imaging [3] and light homogenization [4]. In comparison with conventional single-lenses, MLA allows us to collect rich information. In an integral imaging system, lenslets arranged on the MLA capture a group of sub-images at different spatial positions from different viewing angles. The group of sub-images can be reconstructed together so as to provide the depth information of the scene [5]. In most microlens arrays, the focal length of all the lenslets is identical. This results in a narrow depth of field, making depth perception limited. In addition, when MLA is used for beam homogenization, the incident laser beam is divided into a series of sub-beams, which are superimposed on each other in the far field to eliminate the inhomogeneity between different sub-beams and form a homogenized spot [6,7,8,9,10]. When it is used for high coherence laser beam homogenization, the MLA with the same focal length will produce a periodic lattice phenomenon, resulting in the degradation of homogenization quality and so on. Therefore, researchers have proposed the use of multifocal MLA for the purpose of improving the image quality or beam homogenization effect.

The traditional fabrication methods of MLA are the thermal reflow technique [11,12], laser direct writing (LDW) [13,14,15,16], 3D printing [17,18], and ion irradiation [19,20]. Thermal reflow technology is an effective method of preparing MLA. It involves melting a group of cylindrical photoresist polymers distributed on the substrate into a hemispherical shape so as to obtain a MLA with the same curvature [11]. However, this preparation method can not achieve a MLA with a high filling factor and the preparation of multifocal MLA. Sang-in Bae proposed a new method combining multiple lithography and thermal reflow technology to prepare a multifocal MLA [12]. In this method, multi-layer micro cylinders are constructed by multiple repeated exposure technology, and the multi-layer micro cylinders are thermally refluxed at the same time to realize the fabrication of multifocal MLA. In the fabrication process, the parameters of multifocal MLA can be controlled by the total thickness and diameter of multilayer micro cylinders. However, the fabrication process is cumbersome, and the process of multiple lithography will introduce errors. LDW uses variable intensity laser beams to process optical materials directly or indirectly to form the required relief contour. Daniel Nieto proposed that laser plasma be used for the ablation of glass materials to prepare micro optical elements, and silica coating was deposited on optical elements by sol–gel technology to reduce roughness [13]. Kostyuk, G.K. proposed using laser-induced blackbody heating to rapidly heat the graphite surface to the evaporation temperature to release plasma graphite particles. These particles promote the rapid heating of molten quartz to the melting temperature, thereby changing the glass relief [14]. In addition, Lin, C.H. proposed to modify the microstructure of special glass “foturan” by femtosecond laser direct writing, and then carry out heat treatment, wet etching, and additional annealing to prepare MLA [15]. Two-photon polymerization (TPP) 3D printing can also achieve the fabrication with hundred-nanometer-scale or sub-micrometer-scale resolution, in which two-photon absorption occurring at a focused spot inside the photoresist can trigger a local polymerization and then un-polymerized photoresist can be washed away later [17,18]. Gyula Nagy proposed the use of heavy ion beams to irradiate the surface topography of polydimethylsiloxane along the helical path to prepare MLA with different diameters at one time [19,20]. Using LDW, TPP 3D printing or ion irradiation technique, MLA consisting of lenslets of any desired profile and curvature can be easily realized. Nevertheless, LDW and TPP 3D printing techniques are performed on the basis of point-by-point structural modification and require a long fabrication time for producing large-sized components. Thus, the productivity for producing large-scale components is limited [21].

Xue proposed the combination of chemical wet etching and lithography to prepare multifocal MLA [22]. The lens parameters can be controlled using microporous masks with different sizes and etching time in the lithography process. However, since the control of the focal length and aperture of the lens are interrelated, it is impossible to control the focal length and aperture of each sub-lens unit separately. Recently, the microfluidic manipulation technique has become a new candidate for fashioning optical lenses. In the fabrication of the MLA using the microfluidic manipulation technique, the curvature of the lenslets can be well controlled by harnessing surface tension or applying pressure to reshape the profile of microfluid in the mold [23,24,25,26]. Based on the characteristics of microfluidic technology, Long et al. proposed the preparation of multi-focus MLA with uniform aperture on curved microfluidics with inclined sidewalls. The method uses computer-aided tools to make inclined wall micropores with the same diameter and different inclination angles. Then, a light curing polymer is injected into the micropores, spin coated and exposed to form a curved surface. The MLA with multi focus and uniform aperture was prepared using the micropore as a mold [27], However, this method needs to design each inclined wall angle separately to obtain the multifocal MLA, and the process is cumbersome.

In this paper, the technology of preparing multifocal MLA by a one step exposure process is proposed. The mask of a multifocal MLA will be designed by studying the nonlinear relationship between exposure depth and exposure dose, then the fabrication of multifocal MLA can be realized with the use of mask moving exposure technology [28,29]. The structure of this paper is arranged as follows: Section 2 describes the principle of the fabrication of multifocal MLA, including the forming principle of 3D continuous surface and the design principle of mask. Section 3 demonstrates the nonlinear effect correction of the key process material—photoresist. Section 4 describes the detailed fabrication process and fabrication results, and Section 5 is a summary of the whole paper.

## 2. Fabrication Principle

**Figure 1:**
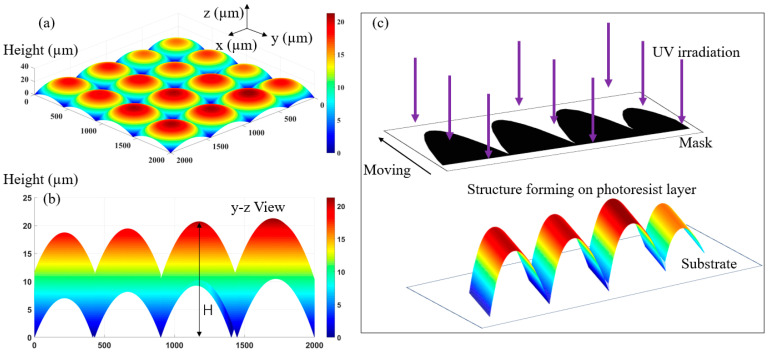
Exposure formation principle of multi-focus MLA: (**a**) 3D profile of MLA; (**b**) Y–Z view; (**c**) Schematic diagram of moving mask exposure.

Due to the difference in the focal lengths of each sub-lens unit in the multifocal MLA, the 3D surface of each sub-lens is different. For example, Figure 1a shows a multifocal MLA designed with our theory. As can be seen from its y–z view (Figure 1b), the sag height of each sub lens unit is different. Because the mask moving exposure technology can realize a fabrication of continuous surface 3D structure, this paper proposes the use of this technology to realize the preparation of multifocal MLA. The basic principle of forming a 3D continuous surface structure by mask moving exposure technology is shown in Figure 1c. A continuous undulating graphic area is designed in the two-dimensional (2D) plane of the mask. The black part is the opaque area while the white part is the transparent area. During the process of exposure, the mask moves a certain distance with the wafer support platform of the lithography machine to realize the continuous adjustment of exposure dose. The photoresist material records the exposure dose of each point transmitted from the light transmission area and realizes the formation of continuous surface structure through process operations such as development and fixing. At this moment, the surface shape of the formed 3D structure is cylindrical, and its height fluctuation is consistent with the pattern of the mask.

**Figure 2:**
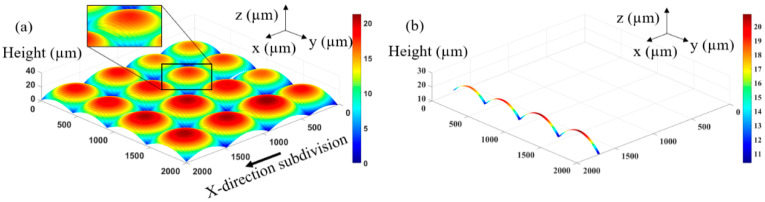
Surface shape of multifocal MLA: (**a**) 3D contour subdivision; (**b**) Spline surface distribution.

Further, in order to realize the spherical surface shape, it is necessary to further analyze its 3D surface shape. The 3D structure of the designed multifocal MLA is subdivided and quantized into a series of equally spaced strip regions according to a certain period T, as shown in Figure 2a. Each strip area can be approximately regarded as a cylindrical lens when the divided period *T* is fine enough, as shown in Figure 2b. Therefore, the surface shape of the overall multifocal MLA can be regarded as composed of several cylindrical lenses with period T, in which period *T* determines the smoothness of the overall surface shape. Inspired by this, the key element used in exposure, that is, the mask, can be designed. The mask will also be composed of equally spaced strip areas with a width of T. The pattern of each strip area in the mask will be designed separately to adjust the exposure dose on the photoresist during exposure to form the corresponding cylindrical lens. All cylindrical lenses are combined to obtain a complete spherical 3D surface.

**Figure 3:**
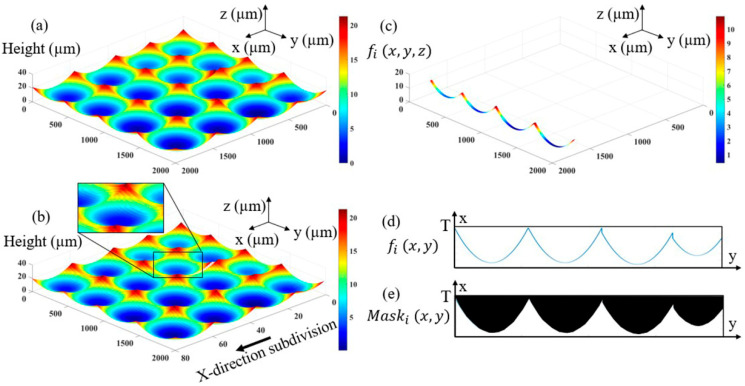
Degumming surface shape: (**a**) 3D contour; (**b**) 3D contour subdivision; (**c**) Spline surface distribution; (**d**) Spline 2D contour; (**e**) Mask cell pattern.

Because the pattern of the strip area on the mask determines the exposure dose at different positions on the photoresist, and thus determines the degumming amount of the photoresist after development, it is necessary to study the degumming amount at different positions of the photoresist according to the 3D shape of the multifocal MLA to design the corresponding mask pattern. The 3D shape of the photoresist material that needs to be removed during exposure and development can be obtained from the 3D shape of the multifocal MLA, as shown in Figure 3a. Similarly, they are subdivided according to the period *T*, as shown in Figure 3b, and the 3D contour of each spline can be obtained. At this time, each spline is a concave cylindrical structure, as shown in Figure 3c. Suppose that the profile function of the 3D structural surface of the spline is $f_{i}(x,y,z)$, where i is the number of the subdivided strip areas, and the division direction is along the *x*-axis. The 3D structure surface shape in the strip area is converted to a 2D curve $f_{i}(x,y)$, as shown in Figure 3d, and its transformation relationship is shown in Equation (1).

(1) $$f_{i}(x,y)=\int_{(i-1)T}^{iT}\frac{f_{i}(x,y,z)}{H_{\max}}dz,i=1,2,\ldots K$$

where K is the total number of subdivisions and $H_{\max}$ the maximum vector height between different sub-lens units in the multifocal MLA.

**Figure 4:**
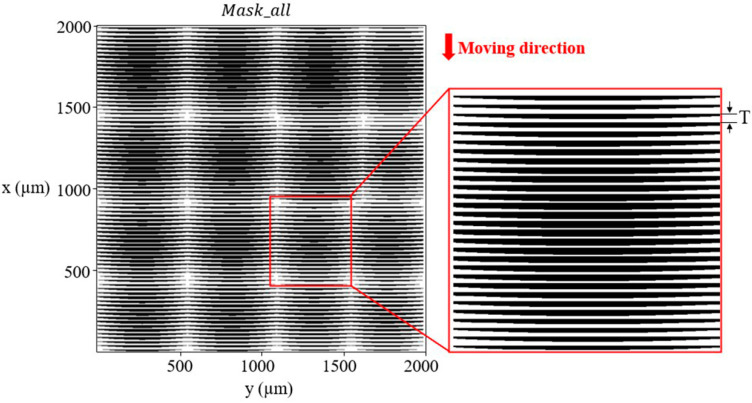
Mask pattern.

Then, the area surrounded by the $f_{i}(x,y)$ and *x*-axis of the 2D curve is set as the light transmitting graphic area, and the graphics of the other areas are opaque to obtain the mask unit pattern $Mask_{i}(x,y)$ of the ith subdivision area, as shown in Figure 3e. Finally, all strip areas in Figure 3b are converted form $f_{i}(x,y,z)$ to $Mask_{i}(x,y)$ one by one. Additionally, all mask unit patterns are combined according to the corresponding position relationship to obtain the final mask structure Mask_all for the overall multifocal MLA, as shown in Figure 4. At this time, it can be roughly observed that the area proportion of the light transmission area between each sub-lens unit is different, and the adjustment of the corresponding exposure dose of sub-lens units with different vector heights is realized. In the exposure process, the moving mask distance is *T*, and the structure is formed by development.

## 3. Nonlinear Effect Correction

Because there is a nonlinear relationship between the degumming amount of photoresist and the required exposure dose during the fabrication of multifocal MLA, nonlinear correction is needed in the design of the mask. During the fabrication of the continuous surface multifocal MLA, the typical positive photoresist AZ9260 with a small absorption coefficient applied to the thick photoresist etching process is selected, and the corresponding exposure process parameters are selected, as shown in Table 1.

At a spin coating speed of 700 rpm and an acceleration of 100 rpm/s, a photoresist thickness of 25 μm is obtained. In order to observe the degumming amount under different exposure doses, that is, the difference of exposure depth, the exposure experiment with the change of exposure dose gradient is designed. Different exposure doses are controlled by successively increasing the exposure time. In the experiment, the exposure time increases from 1 s to 13 s, and the exposure power is 3.5 mW/cm^2^. The binary grating with line width of 2 mm is exposed and the exposure depth after development is tested. After a series of exposures, development and other process steps, the exposure depth data under different exposure doses are tested with a step profilometer (Stylus Profiler System, Dektak XT, Bruker, Karlsruhe, Germany). Each substrate is tested with three groups of data, and its mean value is taken as the average value of exposure depth. At the same time, the stability of the data is described by the standard deviation and confidence interval with a 95% confidence level being given, as shown in Table 2.

Although the nonlinear performance between exposure depth and exposure dose of different types of photoresists is different, it roughly follows the trend of logarithmic function [30,31]. The dose distribution required for achieving the target thickness profile is determined by the contrast curve, which depends on the logarithmic exposure dose required for a normalized photoresist thickness to be removed. The value of the contrast *γ* is defined as the linear slope of the contrast curve, as shown in Equation (2).

(2) $$\begin{array}{l} \gamma =\frac{1}{\ln E_{cl}-\ln E_{th}}=\frac{h\left(x,y\right)/H}{\ln E\left(x,y\right)-\ln E_{th}},\;\\ \mathrm{where}\;0<h\left(x,y\right)<H\;\mathrm{and}\;E_{th}<E\left(x,y\right)<E_{cl} \end{array}$$

where $E_{th}$ represents the critical exposure dose (i.e., the lowest exposure dose of photosensitive reaction of touch light-emitting photoresist), $E_{cl}$ the exposure dose required to completely remove the photoresist layer *H*, and E(x,y) indicates the exposure dose required to achieve an exposure depth of h(x,y).

Equation (2) can be rearranged to obtain Equation (3) in terms of the dose distribution E(x,y) that is required to achieve the desired profile h(x,y). The threshold dose $E_{th}$ and contrast *γ* can be obtained from the above experiment data according to Equation (2).

(3) $$E\left(x,y\right)=\exp\left(\frac{h\left(x,y\right)}{H\cdot \gamma }+\ln E_{th}\right)$$

According to Equation (3), we can obtain the exposure dose E(x,y) required at different exposure depths, so as to obtain the exposure dose distribution corresponding to the design structure.

**Figure 5:**
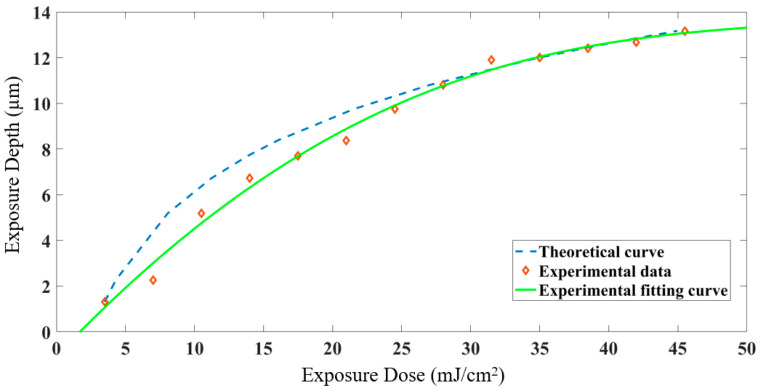
Nonlinear exposure curve.

The correlation fitting analysis is carried out according to Equation (3) and the experimental data in Table 2, and the corresponding theoretical relationship curve and experimental data curve are drawn, as shown in Figure 5. The blue dotted line is the theoretical curve drawn according to Equation (3), and the green solid line is the curve fitted according to the experimental measurement data (diamond symbol). It can be seen that the experimental data are basically consistent with that of the theoretical data, and the relationship between exposure depth and exposure dose follows a logarithmic function. However, the actual processing exposure depth is different from that of the theoretical curve due to the influence of process conditions such as ambient temperature and other parameters. Therefore, the light transmission area distribution of the mask is corrected according to the experimental fitting curve while designing the mask.

**Figure 6:**
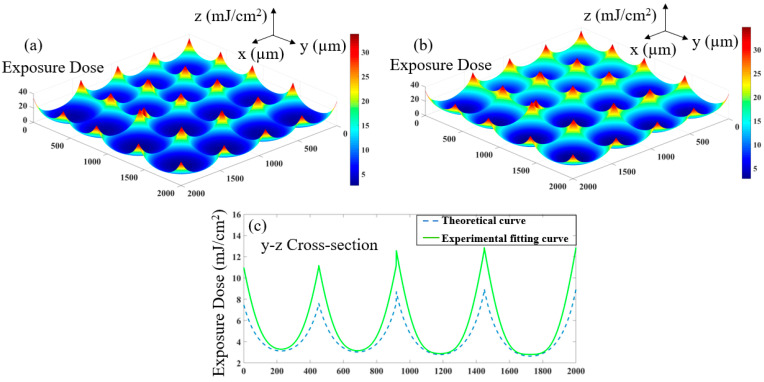
Comparison of degumming amount before and after correction: (**a**) Theoretical 3D distribution of degumming amount; (**b**) Corrected 3D distribution; (**c**) Section comparison before and after correction.

The theoretical exposure dose is corrected according to the nonlinear exposure curve. The 3D distribution of the exposure dose before correction is shown in Figure 6a, and the exposure dose after correction is shown in Figure 6b. In order to clearly distinguish the change in exposure dose before and after correction, we extracted the exposure curve of the same section for comparison, as shown in Figure 6c. It can be seen that more degumming is required in the edge area of the sub-lens unit, and its exposure dose will be increased so as to obtain the target exposure depth. According to this exposure dose distribution function, the 2D curve function $f_{i}(x,y)$ involved in the process of designing the mask is corrected to obtain the corrected $Mask_{i}(x,y)$, and finally to obtain the corrected overall mask Mask_all.

## 4. Fabrication Process, Testing and Characterization

**Figure 7:**
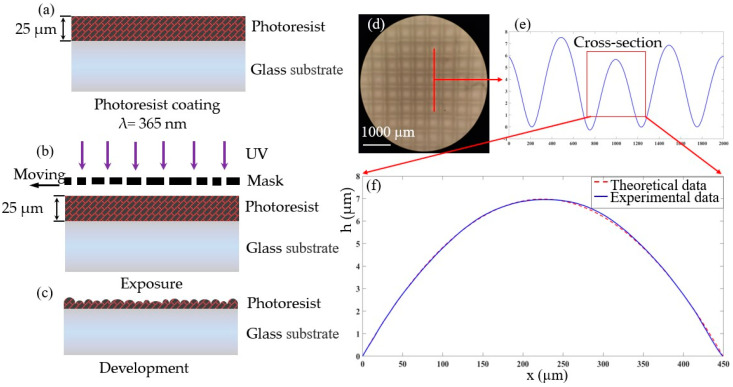
Preparation process and characterization of MLA: (**a**) Spin coated photoresist; (**b**) Moving mask exposure; (**c**) development; (**d**) 2D microstructure; (**e**) Contour representation; (**f**) Zoom in profile of the marked in; (**e**) Comparison between sub-element contour and calculated profile.

Based on the above theoretical study, this section will introduce the fabrication process of multi-focus MLA prepared by one step exposure molding as well as the characterization of the fabrication results. As shown in Figure 7, the fabrication process includes substrate pretreatment, spin coating photoresist, pre baking, exposure, development and post baking. Each link is described in detail below.

Firstly, the substrate is pretreated, the substrate surface is cleaned with acetone, and then dried. Then, the pretreated substrate is spin coated with photoresist using the parameters described in Table 1 in the third section. Secondly, the substrate coated with the photoresist is placed on the hot plate and the temperature is set to 90 °C and the prebaking time is set to 5 min. Thus, the photoresist on the surface of the substrate is cured, as shown in Figure 7a. In the process of prebaking, in order to ensure that the substrate is heated evenly and its surface is not polluted, the thermal insulation cover is covered on the substrate surface. Then, the moving mask is exposed, and the pattern on the mask is transferred to the photoresist, as shown in Figure 7b. The exposure power density of the exposure machine is set to 3 mW/cm^2^, and the central wavelength of the UV light source is 365 nm. During exposure, the moving distance of the mask is 10 μm. That means the subdivision period *T* mentioned in Section 2 is 10 μm. The exposure time is 15 s. Finally, during the development and post-baking, the exposed substrate is put into the diluted (AZ 400k:deionized H_2_O = 1:1) developer for 30 s. At this time, the contour of the continuous relief structure will be generated on the photoresist, and the developed substrate is placed behind the hot plate and baked for 2 min to obtain the structure, as shown in Figure 7c. The prepared structure was magnified and observed by microscope (Olympus BX51). It can be seen from Figure 7d that the surface of the structure is smooth. At the same time, the structure is characterized by a stepped instrument (stylus profiler system, dektak XT, Bruker, Karlsruhe, Germany), as shown in Figure 7e. The variation of fabrication depth and aperture ranges from 8 μm to 16 μm and 450 μm to 550 μm, respectively. According to these geometric parameters of the MLA, the focal length variation range of the lens array is calculated to be from 4 mm to 5 mm. The surface shape of the prepared sub-lens unit is compared with the theoretical spherical surface, as shown in Figure 7f, and we found that the error of the surface shape is within 5%. The results show that the multi-focus MLA can be prepared by the one step exposure process, and the prepared structure has a smooth surface and high pattern fidelity.

## 5. Conclusions

In this paper, the fabrication of multifocal MLA by one step exposure molding is proposed. Through the calibration of photoresist exposure depth and exposure dose, a mask with nonlinear correction is designed and obtained. According to the mask, the MLA is fabricated with an aperture ranging from 450 μm to 550 μm, sag high from 8 μm to 16 μm and the focal length ranging from 4 mm to 5 mm. The results show that the focal length and aperture of the lens can be controlled independently. The arrangement of lenses with specific focal lengths and required apertures in the MLA can be adjusted due to the flexibility of the manufacturing method. We expect that this manufacturing method will be widely used in the production of various imaging and depth sensing applications.

## Figures and Tables

**Table 1:** Exposure process parameters.

Lithography Process	Parameter
Pretreatment of quartz substrate	Wash with acetone and dry
Spin coated photoresist	700 rpm, 100 rpm/s, 25 μm
Pre drying	90 °C, 5 min
Exposure	365 nm, 3.5 mW/cm^2^, 1 s–13 s
Development	AZ 400K: Deionized H_2_O, 1:1, 30 s
Clean	Deionized H_2_O

**Table 2:** Exposure depth data under different exposure doses.

Time of Exposure (s)	Exposure Dose (mJ/cm^2^)	Average Exposure Depth (μm)	Standard Deviation (μm)	Confidence Interval (μm)
1	3.5	1.31	0.02	(1.296,1.324)
2	7.0	2.27	0.10	(2.199,2.341)
3	10.5	5.18	0.07	(5.130,5.230)
4	14.0	6.73	0.07	(6.680,6.780)
5	17.5	7.70	0.08	(7.643,7.757)
6	21.0	8.37	0.02	(8.356,8.384)
7	24.5	9.75	0.08	(9.693,9.807)
8	28.0	10.80	0.03	(10.779,10.821)
9	31.5	11.90	0.11	(11.821,11.979)
10	35.0	12.00	0.08	(11.943,12.057)
11	38.5	12.40	0.08	(12.343,12.457)
12	42.0	12.66	0.06	(12.617,12.703)
13	45.5	13.16	0.04	(13.131,13.189)

## Data Availability

Data will be provided on request through the corresponding author (Axiu Cao) of this article.
